# Mitigating Strain Accumulation in Li_2_RuO_3_ via Fluorine Doping

**DOI:** 10.1021/acs.jpclett.4c00748

**Published:** 2024-05-10

**Authors:** Yanfang Wang, Hongzhi Wang, Yongcong Huang, Yingzhi Li, Zongrun Li, Joshua W. Makepeace, Quanbing Liu, Fucai Zhang, Phoebe K. Allan, Zhouguang Lu

**Affiliations:** † Department of Materials Science and Engineering, Southern University of Science and Technology, Shenzhen, 518055, China; ‡ School of Chemistry, University of Birmingham, Birmingham, B15 2TT, U.K.; § Department of Electronic and Electrical Engineering, Southern University of Science and Technology, Shenzhen, 518055, China; ∥ School of Chemical Engineering and Light Industry, Guangdong University of Technology, Guangzhou, 510006, China

## Abstract

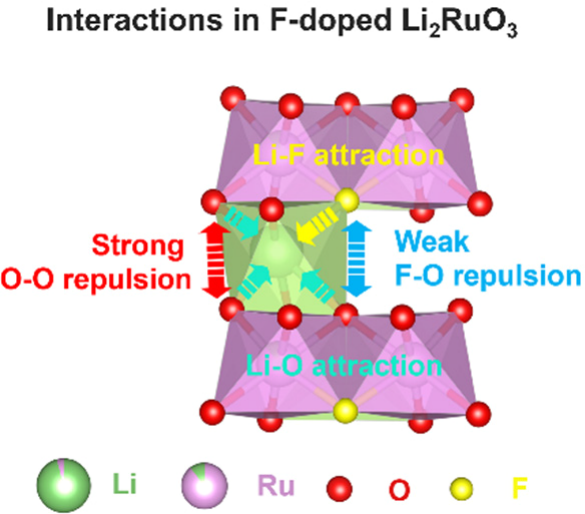

Lithium ruthenium oxide (Li_2_RuO_3_) is an archetypal lithium rich cathode material (LRCM) with both cation and anion redox reactions (ARRs). Commonly, the instability of oxygen redox activities has been regarded as the root cause of its performance degradation in long-term operation. However, we find that not triggering ARRs does not improve and even worsens its cyclability due to the detrimental strain accumulation induced by Ru redox activities. To solve this problem, we demonstrate that F-doping in Li_2_RuO_3_ can alter its preferential orientation and buffer interlayer repulsion upon Ru redox, both of which can mitigate the strain accumulation along the *c*-axis and improve its structural stability. This work highlights the importance of optimizing cation redox reactions in LRCMs and provides a new perspective for their rational design.

Lithium ruthenium oxide (Li_2_RuO_3_, also known as Li[Li_1/3_Ru_2/3_]O_2_), first reported by Dulac in 1970,^1^ has a monoclinic structure (*C*2/*c* or *P*2_1_/*m* symmetry), in which 1/3 of Ru sites in Ru layers are occupied by Li ions to form ordered honeycomb-like patterns (LiRu_6_).^2,3^ However, it was not until two decades later that Li_2_RuO_3_ was revisited thanks to the period enthusiasm of finding suitable layered oxides for lithium de/intercalation. In 1988, James and Goodenough tentatively tried to test its electrochemical properties as the cathode material for lithium-ion batteries, which turned out to be unappealing.^4^ Therefore, in the following two decades, although there were several trials to improve its electrochemical performance via doping or surface coating,^5−7^ Li_2_RuO_3_ was generally out of the interest of electrochemists. Finally, in the 2010s, along with the development of high-voltage electrolytes and the intense research interest in lithium rich cathode materials (LRCMs), Li_2_RuO_3_ regained the spotlight and soon became an archetypal model to investigate the underlying mechanisms of oxygen redox activities.^8^

Previous studies on Li_2_RuO_3_ have revealed that, upon the removal of the first Li^+^, the Ru^4+^-to-Ru^5+^ oxidation is responsible for charge compensation, during which the monoclinic phase (Li_2_RuO_3_, *C*2/*c* or *P*2_1_/*m*) transforms into the rhombohedral phase (Li_1.0_RuO_3_, *R*3̅).^9^ Removing the second Li^+^ triggers the oxidation of lattice oxygen (O^2–^) and induces O–O dimerization, producing super/peroxide like species (O_2_^*n*–^, 0 < *n* ≤ 2) and even molecular O_2_.^10,11^ Commonly, to form O–O dimers, the decoordination between Ru and O is inevitable, which promotes irreversible Ru migration and oxygen loss.^12^ Therefore, the structural instability induced by oxygen redox activities has been widely regarded as the origin of its long-term performance degradations, i.e., capacity loss and voltage decay.^13^

However, we find that not triggering oxygen redox activities does not guarantee better long-term stability of Li_2_RuO_3_. On the contrary, solely exploiting Ru redox reactions unexpectedly worsens its cycling performance. Such a phenomenon is suggested to originate from the important yet overlooked strain accumulation induced by Ru redox activities. On the one hand, upon Ru^4+^-to-Ru^5+^ oxidation, removing Li^+^ ions diminishes their screening effects and results in stronger electrostatic repulsion between O layers. On the other hand, because it undergoes a biphasic process upon Ru oxidation, nanoscale strain and lattice displacement can emerge at phase boundaries in delithiated Li_2–*x*_RuO_3_ (0 < *x* < 1). Such heterogeneities have been proven to be driving forces of structural degradation in many cathode materials.^14,15^ In this study, considering that the strain-induced structural degradation is basically a bulk issue and doping is a potential way out of this, we demonstrate that F-doping can effectively improve the stability of Ru redox activities in Li_2_RuO_3_. Specifically, F-dopants replace O ions, meaning that the weaker O–F repulsion favors smaller lattice expansion along the *c*-axis upon delithiation. Moreover, F-doping alters the preferential orientation so that the strain accumulation can be relaxed.

First, pristine Li_2_RuO_3_ and F-doped Li_1.83_RuO_2.83_F_0.17_ were prepared via solid-state reactions (Experimental Section). As shown in the X-ray photoelectron spectroscopy (XPS) results (Figure S1), the positions of the Ru 3p peaks are the same in both samples (i.e., 486.3 and 464.2 eV for 3p1/2 and 3p3/2, respectively), which implies that Ru is in the valence state of 4+.^8^ Peaks at around 529.8 eV in O 1s spectra can be assigned to lattice oxygen (O^2–^), while those at higher binding energies are from surface adsorbents.^10^ In the F 1s XPS spectra, no peak is observed in pristine Li_2_RuO_3_, while the peak at around 684.4 eV in F-doped Li_1.83_RuO_2.83_F_0.17_ can be assigned to a Li–F or Ru–F bond,^16^ indicating that F anions are successfully embedded into the lattice. In addition, depth profiling shows that such a peak remains after Ar^+^ beam etching, meaning that F-dopants also exist in the bulk (Figure S2). In addition, according to elemental mappings (Figure S3), no F is observed in pristine Li_2_RuO_3_, whereas it disperses uniformly in F-doped Li_1.83_RuO_2.83_F_0.17_.

The effects of F-dopants are carefully analyzed via Rietveld refinements of their synchrotron X-ray diffraction (XRD) patterns. For pristine Li_2_RuO_3_, without considering Li/Ru mixing and preferred orientation, it matches quite well with an ideal *P*2_1_/*m* model with an *R*_wp_ of 14.45% and a G.O.F. of 1.57, though some peaks are strong while some others are weak, implying that it has a certain preferred orientation (Figure S4 and Table S1).^2^ After taking the preferred orientation into consideration, wherein sixth order spherical harmonics were used, the refinement was further improved with an *R*_wp_ of 11.02% and a G.O.F. of 1.20 (Figure S5 and Table S2). Finally, the intralayer Li/Ru mixing was refined and it improved the refinement slightly, i.e., an *R*_wp_ of 10.70% and a G.O.F. of 1.16 (Figure 1a and Table S3). The quantity of intralayer Li/Ru mixing is about 2.36%. For F-doped Li_1.83_RuO_2.83_F_0.17_, its XRD patterns also fit well with a *P*2_1_/*m* model without intralayer Li/Ru mixing and preferred orientation, while the values of *R*_wp_ and G.O.F. are 14.13% and 2.24, respectively (Figure S6 and Table S4). After considering the preferred orientation, those values largely decrease to 9.92% and 1.57, respectively (Figure S7 and Table S5). When both the preferred orientation and intralayer Li/Ru are refined, they further decrease to 8.80% and 1.39, respectively (Figure 1b and Table S6). In the final structure, the quantity of intralayer Li/Ru mixing is about 13.3%. Therefore, F-dopants in Li_1.83_RuO_2.83_F_0.17_ increase the ratio of intralayer Li/Ru mixing and alter its preferential orientation. In addition, F-doping induces anisotropic lattice distortions, i.e., 0.93% expansion along the *a*-axis, 0.31% contraction along the *b*-axis, and 0.04% shrinkage along the *c*-axis.

**Figure 1:**
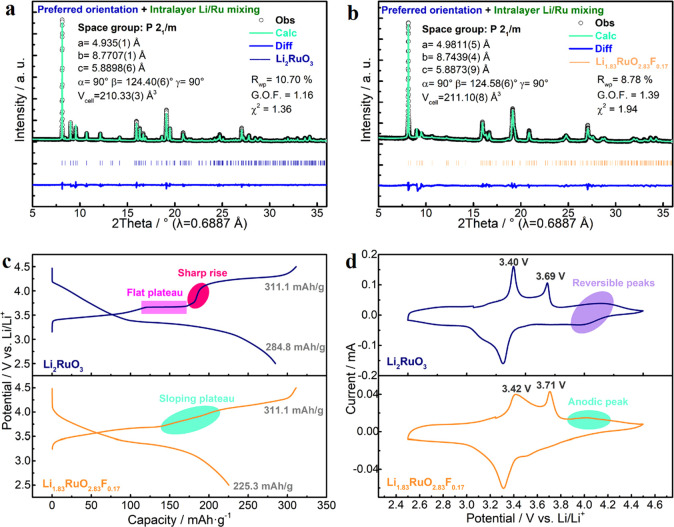
Rietveld refinements of (a) Li_2_RuO_3_ and (b) Li_1.83_RuO_2.83_F_0.17_ with synchrotron X-ray diffraction (XRD) patterns (λ = 0.6887 Å). (c) The 1st galvanostatic charge–discharge (GCD) curves (20 mA/g, 2.5–4.5 V vs Li/Li^+^) and (d) the 2nd cyclic voltammetry curves (0.05 mV/s, 2.5–4.5 V vs Li/Li^+^) of Li_2_RuO_3_ and Li_1.83_RuO_2.83_F_0.17_ electrodes.

When tested as the cathode material, the Li_2_RuO_3_ electrode presents typical voltage profiles with an initial charging capacity of 311.1 mAh/g (Figure 1c, 20 mA/g, 2.5–4.5 V vs Li/Li^+^), namely, a slope at around 3.5 V, a flat plateau at 3.67 V, and a slope above 3.9 V vs Li/Li^+^.^17^ Specifically, the first two processes are attributed to two periods of Ru redox reactions.^18^ By contrast, given that the theoretical capacity upon Ru^4+^-to-Ru^5+^ oxidation is 164 mAh/g, the extra capacity contributed by the slope above 3.9 V vs Li/Li^+^ originates from oxygen oxidation. It is noteworthy that the sharp potential rise to trigger oxygen redox activities indicates the large energy gap between energy bands of Ru and O.^19^ In discharge, it delivers a capacity of 284.8 mAh/g, i.e., an 8.5% capacity loss. Meanwhile, the initial staircase-like charging profile disappears in the first discharge and the following cycles, resulting from irreversible structural rearrangement (Figure S8a).^20^ Moreover, such a structural evolution lowers the potential to activate ARRs, consistent with the leftward shift of the anodic peak in cyclic voltammetry (CV) curves (Figure S8b). As to the Li_1.83_RuO_2.83_F_0.17_ electrode, although it delivers a comparable capacity of 311.1 mAh/g in the first charge (20 mA/g, 2.5–4.5 V vs Li/Li^+^), F-doping clearly changes its electrochemistry (Figure 1c, Figure S8c and S8d). Specifically, in its first galvanostatic charge–discharge (GCD) curves, a sloping plateau rather than a sharp potential rise bridges cation and anion redox processes. To understand this, the higher level of local disordering (i.e., Li/Ru mixing) might play part of the role as it favors the gradual phase transformation, vide infra.^21^ In addition, F-doping can alter O 2p and Ru 4d orbitals and increase the overlap between O and Ru bands.^22^ Consequently, as presented in Figure 1d, anodic peaks of Ru and O oxidations shift to higher and lower potentials, respectively. Notwithstanding the liability of facilitating ARRs, F-doping deteriorates the reversibility of oxygen redox as the Li_1.83_RuO_2.83_F_0.17_ electrode bears a 27.6% capacity loss in the first discharge (85.8 mAh/g).

Upon cycling, the Li_2_RuO_3_ electrode delivers initial capacities of 284.8, 186.3, and 146.9 mAh/g and capacity retentions of 62.0%, 75.2%, and 54.7% after 100 cycles, within the potential windows of 2.5–4.5, 2.5–4.2, and 2.5–3.9 V vs Li/Li^+^, respectively (Figures S9–S12). Considering that both cation and anion redox reactions can be fully activated upon charging to 4.5 V vs Li/Li^+^, the rapid capacity decay can be attributed to known irreversible oxygen loss and structural degradation.^23^ However, the even worse cyclability with the upper limit potential of 3.9 V vs Li/Li^+^ cannot be blamed on the instability of oxygen redox activities because they are supposed to be untriggered or limited. Such an inferior stability might be attributed to the accumulation of strong interlayer repulsions and stacking faults caused by the incomplete phase transformation.^23^ As to the F-doped Li_1.83_RuO_2.83_F_0.17_ electrode, it shows rapid capacity decays within the potential windows of 2.5–4.5 and 2.5–4.2 V vs Li/Li^+^ (i.e., 48% capacity retention for both after 100 cycles), though F-doping energetically favors the oxygen oxidation, meaning that striking the balance between liability and stability of oxygen redox activities is still a dilemma. However, it displays a much better cyclability within the potential window of 2.5–3.9 V vs Li/Li^+^ (i.e., 84% capacity retention after 100 cycles), suggesting that F-doping is effective in improving the Ru redox stability in Li_2_RuO_3_.

To study the effects of F-doping on structural evolutions, in situ XRD tests were performed for both pristine Li_2_RuO_3_ and Li_1.83_RuO_2.83_F_0.17_ electrodes within the potential window of 2.5–3.9 V vs Li/Li^+^. For pristine Li_2_RuO_3_ (Figure 2a), during the first charging slope at around 3.5 V, the initial monoclinic phase (named P1 here) transforms to another monoclinic phase (named P2 here), in which the interlayer spacing along the *c*-axis increases as the (001) peak shifts leftward from 2θ = 18.16° to 17.99° with a Δ2θ of 0.17°. The P1-to-P2 phase transformation is complete before the flat plateau at 3.67 V. Upon further delithiation, the monoclinic P2 phase gradually converts to a rhombohedral phase (named P4 here) with an intermediate phase (named P3 here).^18^ On discharge, the P2 phase reforms before the P4 phase is completely transformed to the P3 phase. Further lithiation leads to the rightward shifting of the (001) peak and the P2-to-P1 phase transformation. Although the P1 phase recovers at the end of lithiation, its interlayer spacing across the *c*-axis is larger than that in the pristine state as the (001) peak is at 2θ = 18.12°, suggesting that the structural evolution of pristine Li_2_RuO_3_ is not fully reversible. In the second charge, similar processes as those in the first charge are observed. During the full charge–discharge process, the largest change of the first peak (Δ2θ) is 0.87°.

**Figure 2:**
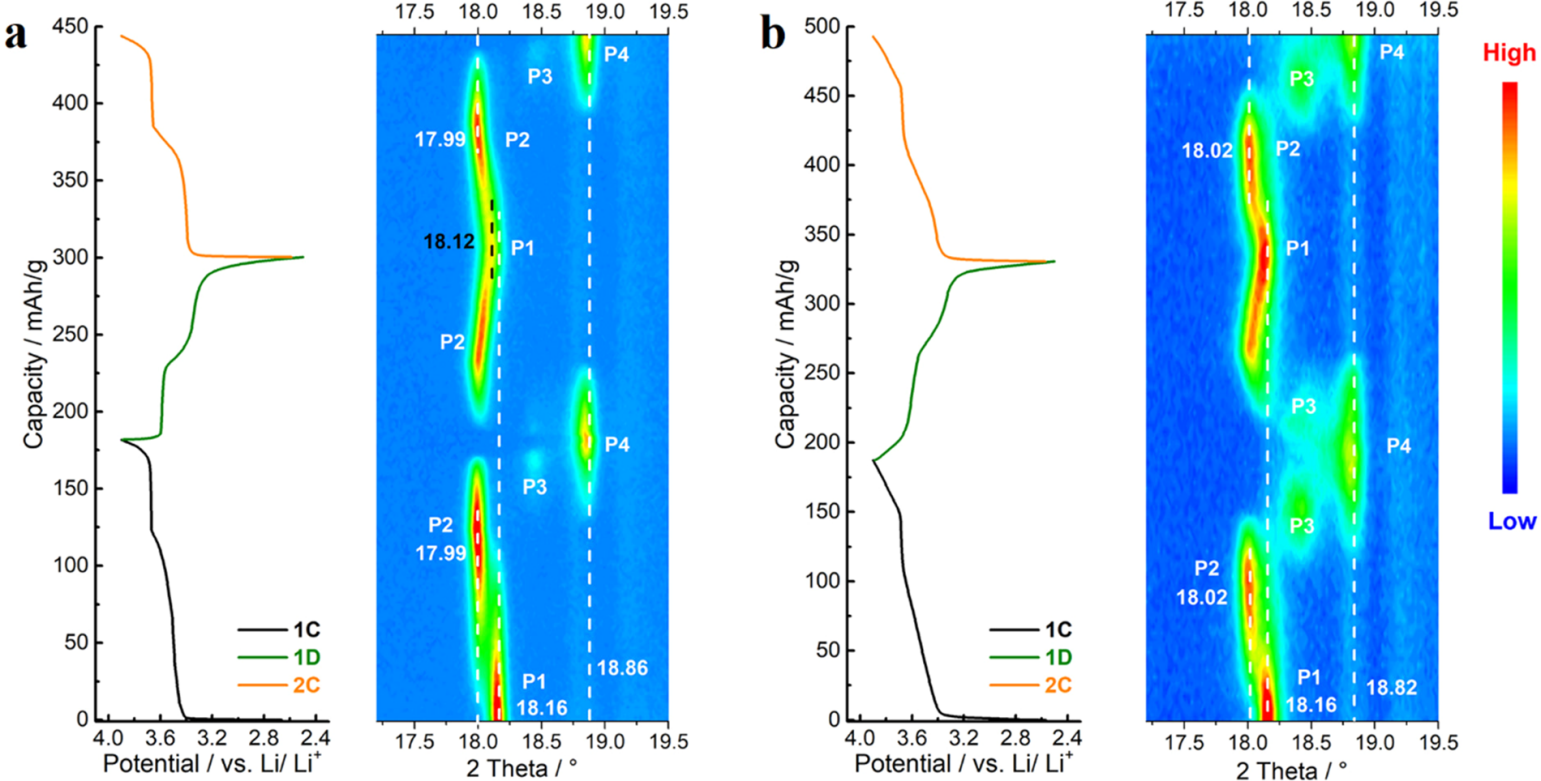
In-situ XRD results of (a) pristine Li_2_RuO_3_ and (b) Li_1.83_RuO_2.83_F_0.17_ electrodes (Cu K_α_, λ = 1.54 Å). The GCD curves are displayed on the left, while the right parts are contour maps of in situ XRD patterns. The potential window is 2.5–3.9 V vs Li/Li^+^, and the current density is 10 mA/g.

For F-doped Li_1.83_RuO_2.83_F_0.17_ (Figure 2b), upon de/lithiation, it undergoes similar phase transformation processes as those in the pristine Li_2_RuO_3_. The (001) peak shifts from 2θ = 18.16° to 18.02° during the P1-to-P2 phase transformation with a smaller Δ2θ of 0.14°. In addition, the intermediate P3 phase is clearly observed in both the P2-to-P4 and P4-to-P2 processes upon delithiation and lithiation, respectively. At the end of lithiation, the (001) peak of the P1 phase almost returns to the original position (2θ = 18.16°), indicating highly reversible structural evolution. During the full charge–discharge process, the largest change of the first main peak (Δ2θ) is 0.80°, smaller than that in the pristine Li_2_RuO_3_. Accordingly, the F-doped electrode shows smaller interlayer spacing changes than those of the pristine Li_2_RuO_3_ electrode during both the P1-to-P2 phase transformation and the full de/lithiation processes, thereby favoring its better long-term stability upon cycling.

To further verify the effectiveness of F-doping, Li_1.95_RuO_2.95_F_0.05_ was prepared, in which only 1.7% O sites are occupied by F dopants. Morphologically, it is composed by polyhedral particles with sizes of several hundred nanometers (Figure S13), while all elements disperse uniformly within single particles (Figure 3a), manifesting successful F-doping. Rietveld refinement of its synchrotron XRD patterns reveals the increasing ratio of intralayer Li/Ru mixing (22.9%) and indicates a similar anisotropic unit cell expansion, i.e., 2.21% expansion along the *a*-axis, 0.53% contraction along the *b*-axis, and 0.11% shrinkage along the *c*-axis (Figure 3b and Table S7). Meanwhile, both E_g_ and A_1g_ mode Raman peaks redshift to lower wavenumbers, suggesting easier Ru–O vibrations due to the F-induced local distortion (Figure 3c).^24^

**Figure 3:**
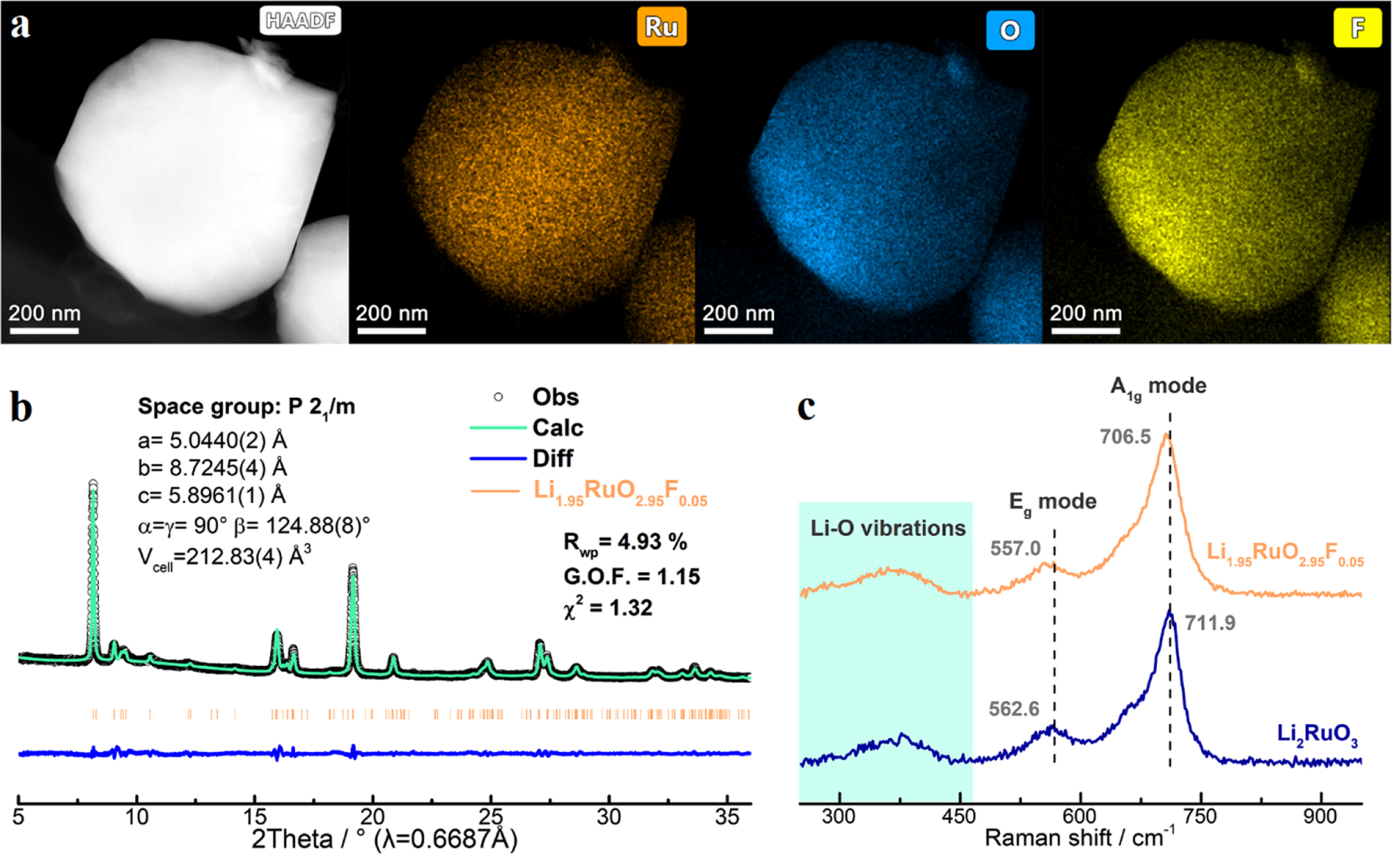
(a) High-angle annular dark-field-scanning transmission electrode microscope (HAADF-STEM) images and elemental mappings of Li_1.95_RuO_2.95_F_0.05_. (b) Rietveld refinements of Li_1.95_RuO_2.95_F_0.05_ with synchrotron XRD patterns (λ = 0.6887 Å). (c) Raman spectra of Li_2_RuO_3_ and Li_1.95_RuO_2.95_F_0.05_.

As displayed in Figure 4a, the Li_1.95_RuO_2.95_F_0.05_ electrode presents liable oxygen redox activities as the sloping plateau at around 3.9 V vs Li/Li^+^ indicates the concurrent Ru and O redox reactions. The sharp anodic peak in d*Q*/d*V* curves implies irreversible structural rearrangement, like that of the undoped Li_2_RuO_3_ electrode. When cycled in the potential window of 2.5–3.9 V vs Li/Li^+^, its capacity decreases from 149.5 to 127.0 mAh/g after 5 cycles, while the Coulombic efficiency (C.E.) increases from 82.8% to 99.0% (Figure 4b). Such capacity loss might originate from irreversible oxygen redox activities promoted by F-dopants because the capacity of the Li_2_RuO_3_ electrode even increases in the initial five cycles (i.e., from 146.9 to 155.1 mAh/g). Upon further cycling, the capacity of the Li_2_RuO_3_ electrode rapidly decays to 25.1 mAh/g after 325 cycles (i.e., 16.2% capacity retention) accompanied by the drop of C.E. to lower than 95.0%. However, for the Li_1.95_RuO_2.95_F_0.05_ electrode, it displays superb long-term stability after the initial activation, i.e., 91.4% capacity retention after 439 cycles with C.E. higher than 99.8% and stable d*Q*/d*V* peaks (Figure S14).

**Figure 4:**
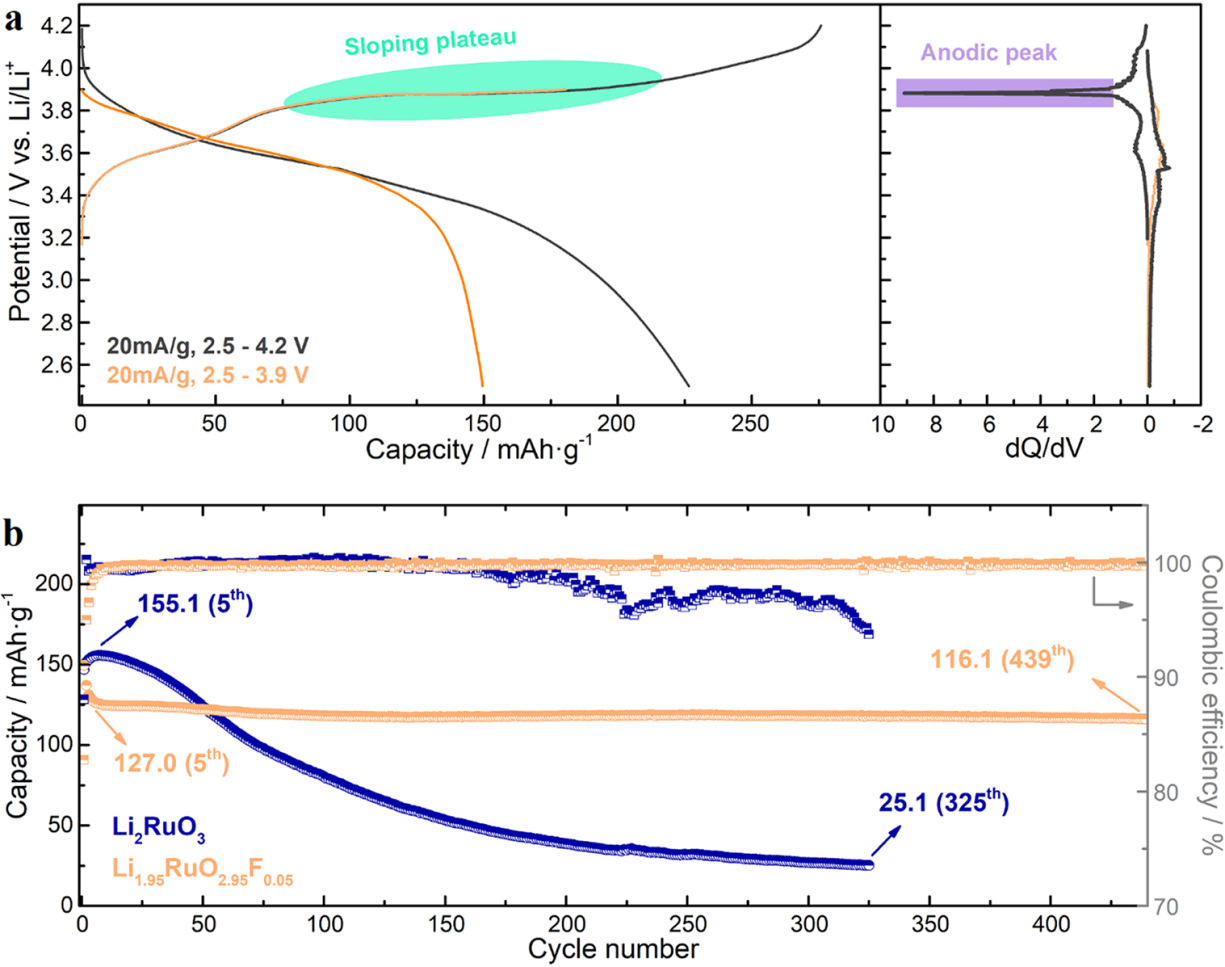
(a) GCD (left) and d*Q*/d*V* curves (right) of the Li_1.95_RuO_2.95_F_0.05_ electrode within potential windows of 2.5–3.9 and 2.5–4.2 V vs Li/Li^+^. The current density is 20 mA/g. (b) Cycling performances of Li_2_RuO_3_ and Li_1.95_RuO_2.95_F_0.05_ electrodes at 20 mA/g within the potential window of 2.5–3.9 V vs Li/Li^+^.

To understand the roles of F-dopants, Figure S15 depicts the interlayer interactions in the pristine and F-doped Li_2_RuO_3_ upon Ru redox reactions. Generally, in the pristine Li_2_RuO_3_, the interlayer spacing is determined by the attractive Li–O and repulsive O–O interactions. Upon Ru oxidation, removing Li^+^ ions weakens their screening effect, while the strong O–O repulsion drives lattice expansion and induces nanostrains along the *c*-axis.^25^ Upon cycling, to relax the strain accumulation, nanocracks form inevitably, resulting in particle pulverization and capacity decay.^26−28^ By contrast, in the F-doped material, despite the loss of the counteracting Li–O attraction upon delithiation, having F-dopants on O sites diminishes the repulsive interaction between the O layers. Therefore, both the weak F–O repulsion and the altered preferential orientation can mitigate the strain accumulation along the *c*-axis, thereby suppressing the formation of nanocracks along the basal plane (*ab*-plane).

In summary, we found that irreversible oxygen redox reaction was not the only origin of capacity decay and voltage fading in Li_2_RuO_3_. By contrast, it displays the worst capacity retention upon solely Ru redox activities, which is proposed to originate from the strain accumulation during the removal of the first Li^+^. To solve this problem, we have demonstrated that F-doping is effective via (i) buffering strong repulsion between the O layers, (ii) inducing local disordering, and (iii) suppressing the preferential orientation along the *c*-axis. Admittedly, although F-doping improves the stability of Ru redox reactions in Li_2_RuO_3_, it deteriorates its stability when oxygen redox reactions are exploited. Therefore, albeit promising, taking advantage of both cation and anion redox reactions in LRCMs is challenging. Looking forward, considering the complexity of their charge compensation and structural evolution mechanisms (e.g., various redox activities and multiple phase transformations), multiangle strategies should be explored to synergistically improve properties of LRCMs.

## Supporting Information Available

Experimental Section detailing synthesis, electrochemical tests, and characterizations; SEM images; XRD patterns; XPS spectra; Rietveld refinement results; CV, GCD, and d*Q*/d*V* results; and schematic illustrations (PDF)
