# Supplementary material for: Mitigating Strain Accumulation in Li2RuO3 via Fluorine Doping
Source: J Phys Chem Lett. 2024 May 10;15(20):5359–65. doi: 10.1021/acs.jpclett.4c00748 (PMC11129289; doi:10.1021/acs.jpclett.4c00748)
Supplement: Supplementary file 1 — jz4c00748_si_001.pdf [file jz4c00748_si_001.pdf]

Mitigating Strain Accumulation in Li_2RuO_3 via Fluorine Doping

Yanfang Wang,[†, ‡, ‖] *Hongzhi Wang,*[†] *Yongcong Huang,*[†] *Yingzhi Li,*[†] *Zongrun Li,*[†] *Joshua W. Makepeace,*[‡] *Quanbing Liu,*[□] *Fucai Zhang,*[*, ‖] *Phoebe K. Allan,*[*, ‡] *and Zhouguang Lu,*[*, †]

[†]Department of Materials Science and Engineering, Southern University of Science and Technology, Shenzhen, 518055, China

[‡]School of Chemistry, University of Birmingham, Birmingham, B15 2TT, UK

[‖]Department of Electronic and Electrical Engineering, Southern University of Science and Technology, Shenzhen, 518055, China

[□]School of Chemical Engineering and Light Industry, Guangdong University of Technology, Guangzhou, 510006, China

Experimental Section

Supplementary Figures S1-S15

Supplementary Tables S1-S7

Supplementary References

Experimental Section

Synthesis

All samples were prepared via traditional solid-state reactions. To prepare Li_2RuO_3, stoichiometric amounts of RuO_2 (Sigma-Aldrich, 99.99 %) and Li_2CO_3 (Sigma-Aldrich, 99.0 %) were mixed and ground by hand. The mixture was then transferred into a muffle furnace and annealed at 450 °C for 4 h (5 °C/ min) and then at 1000 °C for 12 h (2 °C/ min), and gradually cooled to 100 °C (2 °C/ min). To prepare $Li_{1.83}RuO_{2.83}F_{0.17}$, stoichiometric amounts of RuO_2, Li_2CO_3 and LiF (Sigma-Aldrich, 99.9 %) were mixed and ground by hand. The mixture was annealed following similar processes as above, and the final annealing temperatures was 900°C. To prepare $Li_{1.95}RuO_{2.95}F_{0.05}$, stoichiometric amounts of RuO_2, Li_2CO_3 and LiF were mixed and ball-milled in acetone. The mixture was dried in vacuum oven at 120 °C for 12 h, transferred into a muffle furnace and annealed at 450 °C for 4 h (5 °C/ min) and then annealed at 875°C for 12 h (2 °C/ min), and gradually cooled to 100 °C (2 °C/ min).

Characterization

The morphology of the as-prepared materials was characterized by using scanning electron microscope (SEM) (TESCAM MIRA 3) and transmission electron microscope (TEM) (Talos F200X). X-ray photoelectron spectroscopy (XPS) data were collected on a PHI5300 X-ray photoelectron spectrometer using Mg K_α radiation (250 W, 14 kV). In-situ XRD tests were performed on a Rigaku X-ray diffractometer (Rigaku, Japan) with a Cu K_α radiation (λ=1.54 Å, 9 kW) and a one-dimensional detector was used to increase the intensity of diffractive signals. Synchrotron XRD patterns were collected at beamline 14B of Shanghai Synchrotron Radiation

Facility (SSRF). Raman spectra were performed on a Renishaw inVia Raman spectrometer with a green laser (532 nm). GSAS-II (Py-GSAS) was used for Rietveld refinements.[1]

Electrochemical measurement

The as-prepared materials, carbon black and polyvinylidene fluoride (PVDF) in the weight ratio of 8: 1: 1 were mixed homogeneously in N-methyl pyrrolidone (NMP) and coated on an aluminum foil (12 μm in thickness) before being cut into pieces (1.2 cm in diameter). CR2016-type coin cells were assembled in an argon-filled glovebox by using the as-prepared electrodes (cathode, ~50 μm in thickness), lithium metal plates (anode, 200 μm in thickness) and Celgard membranes (separator, 25 μm in thickness). The areal mass loading of active material is around 2.0 mg/ cm^2. The amount of electrolyte injected for each coin-cell was around 40 μL in volume. The electrolyte was a solution of 1 M $LiPF_6$ in ethylene carbonate (EC)/ dimethyl carbonate (DMC) binary solvent (1:1 in volume). Cyclic voltammetry (CV) and galvanostatic charge-discharge (GCD) tests were performed on a BioLogic electrochemical workstation. Long-term cycling performances were tested on a Neware battery cycler (CT-4008-5V-10mA-164, Shenzhen, China). All tests were performed at 25 °C.

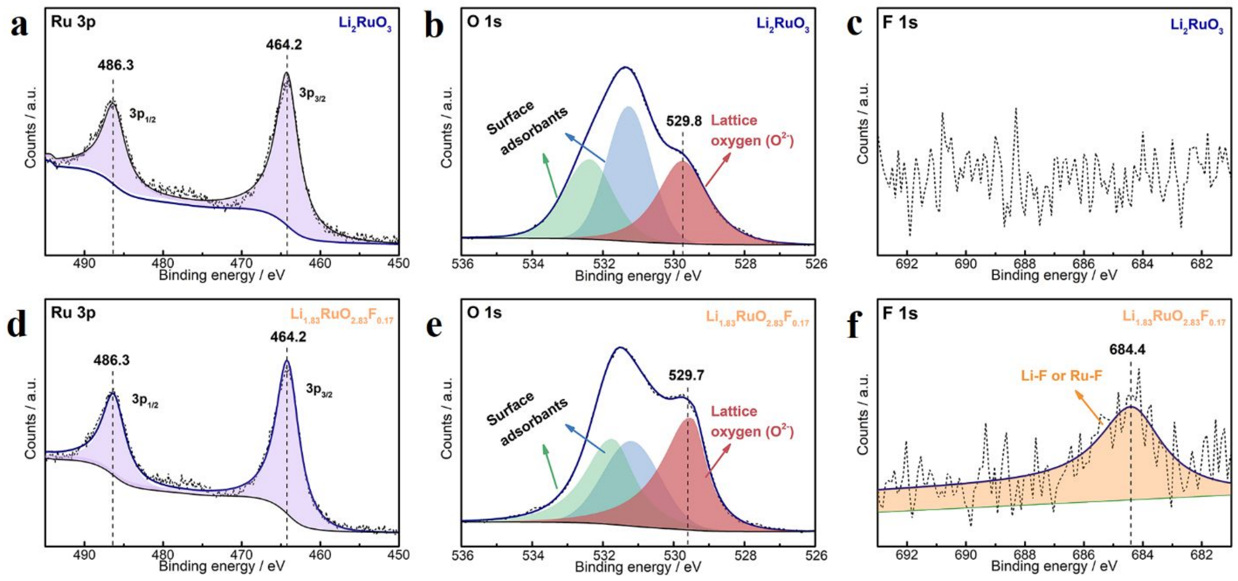

Figure S1. (a) Ru 3p, **(b)** O 1s and **(c)** F 1s XPS spectra of Li_2RuO_3. **(d)** Ru 3p, **(e)** O 1s and **(f)** F 1s XPS spectra of $Li_{1.83}RuO_{2.83}F_{0.17}$. All peaks were corrected based on the C 1s peak (284.8 eV).

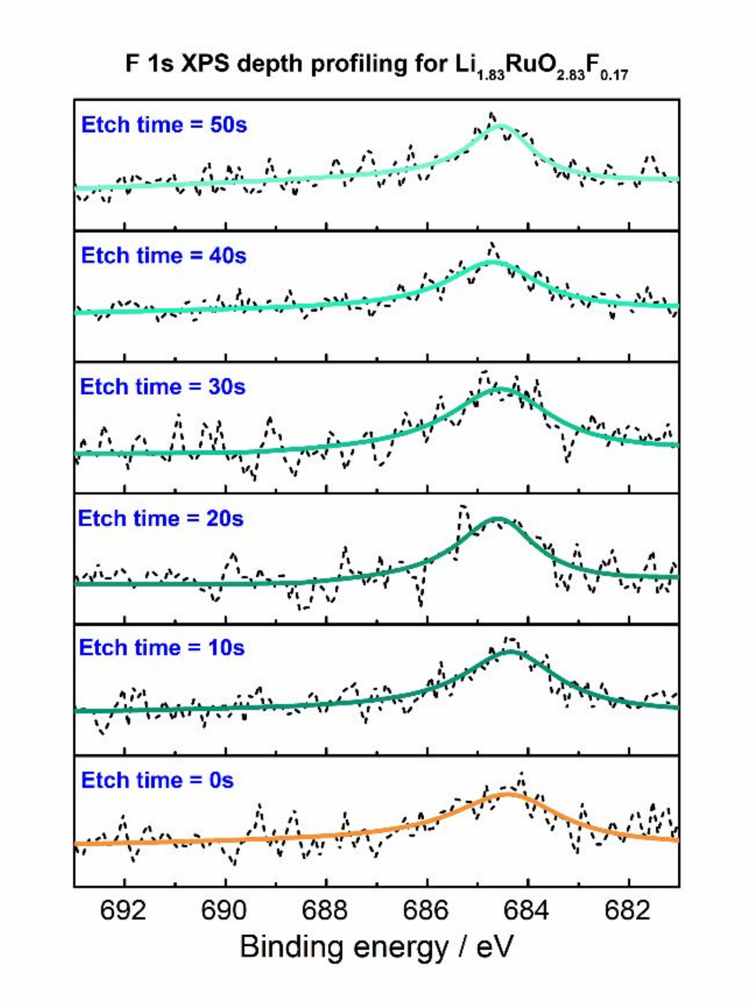

Figure S2. Depth profiling of F 1s XPS spectra of $Li_{1.83}RuO_{2.83}F_{0.17}$. The etching speed of Ar^+ beam is around 0.74 nm/s.

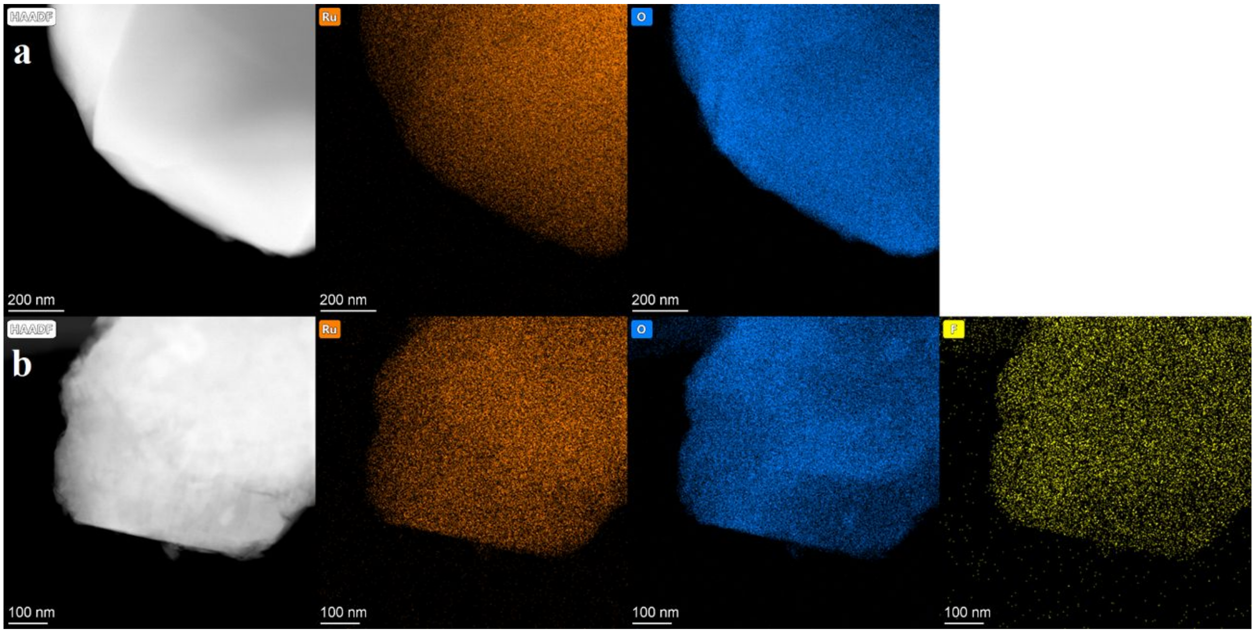

Figure S3. High-angle annular dark field-scanning transmission electrode microscope (HAADF-STEM) images and elemental mappings of **(a)** Li_2RuO_3 and **(b)** $Li_{1.83}RuO_{2.83}F_{0.17}$.

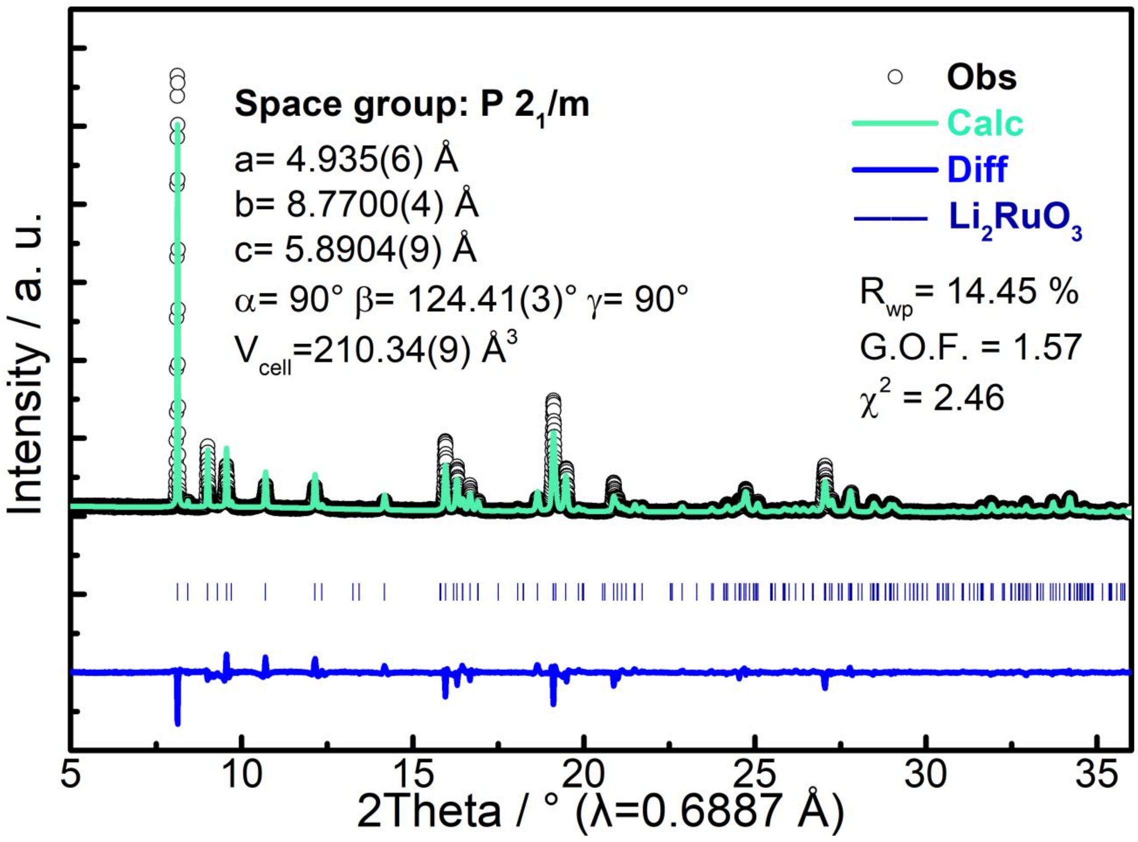

Figure S4. Rietveld refinement of Li_2RuO_3 with synchrotron XRD patterns (λ=0.6887 Å) without considering Li/Ru mixing and preferred orientation.

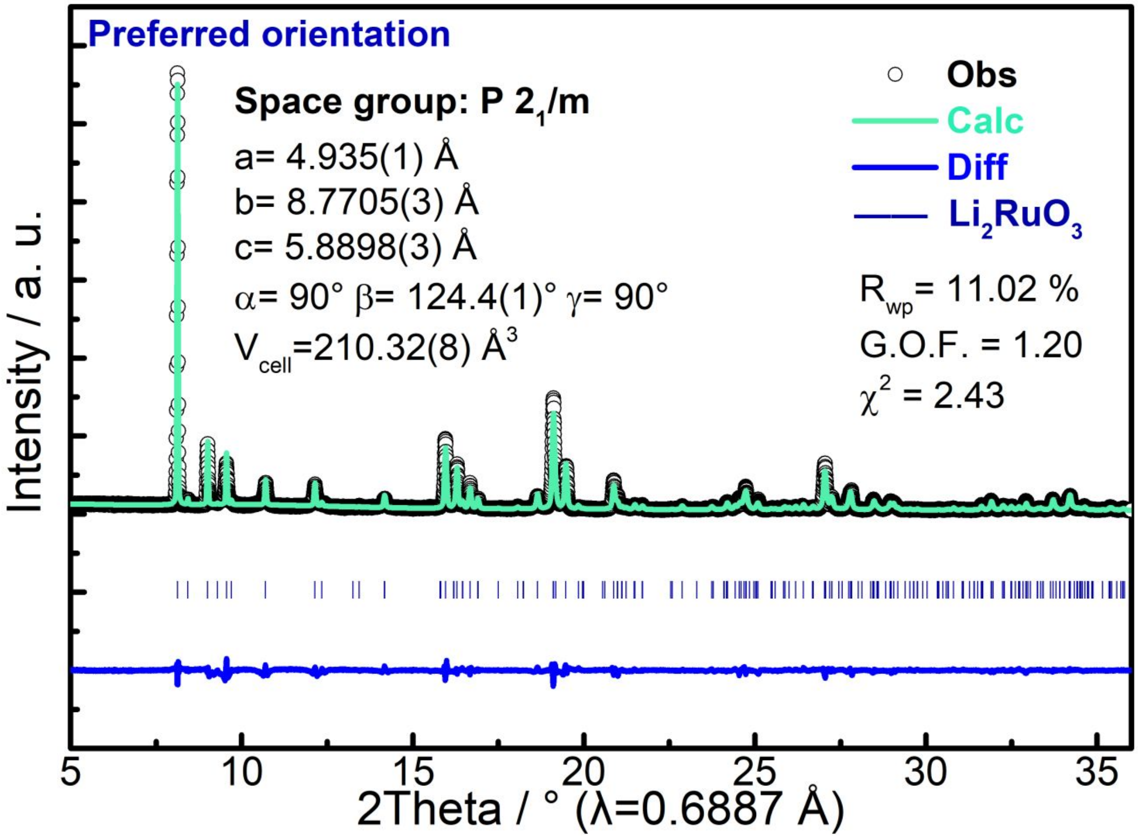

Figure S5. Rietveld refinement of Li_2RuO_3 with synchrotron XRD patterns (λ=0.6887 Å) with preferred orientation considered.

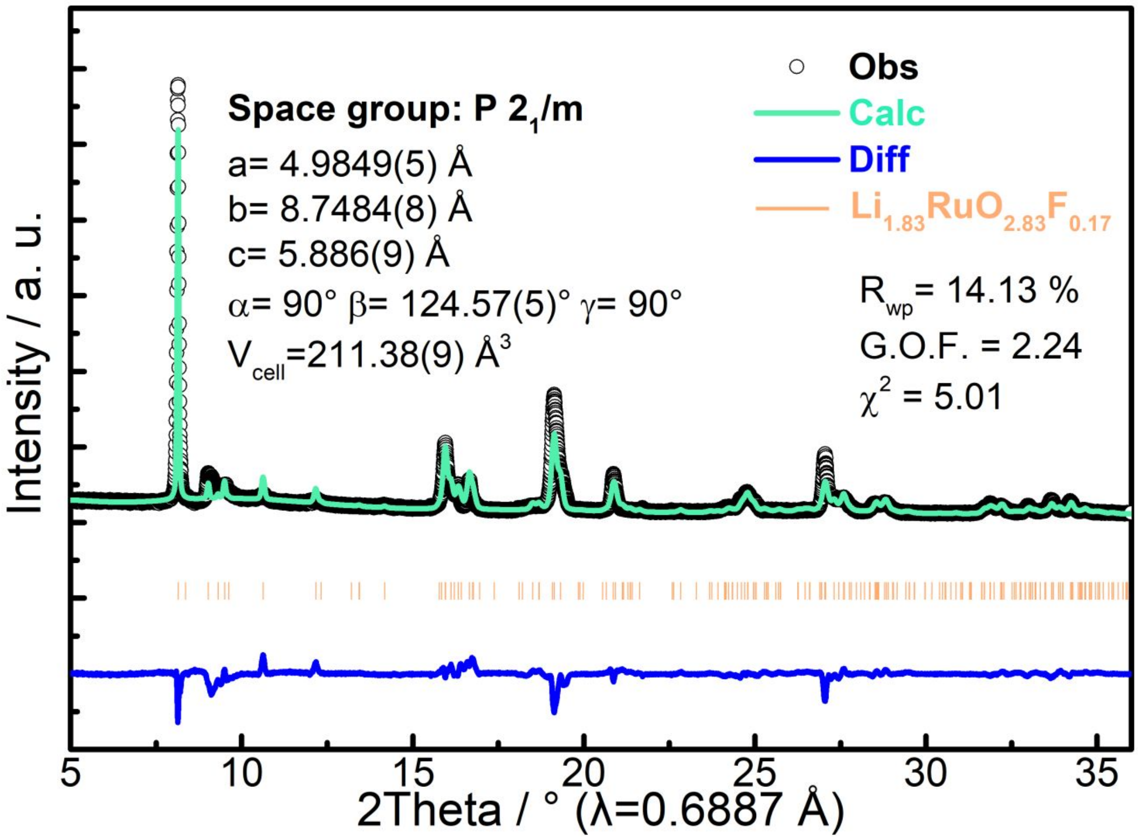

Figure S6. Rietveld refinement of $Li_{1.83}RuO_{2.83}F_{0.17}$ with synchrotron XRD patterns (λ=0.6887 Å) without considering Li/Ru mixing and preferred orientation.

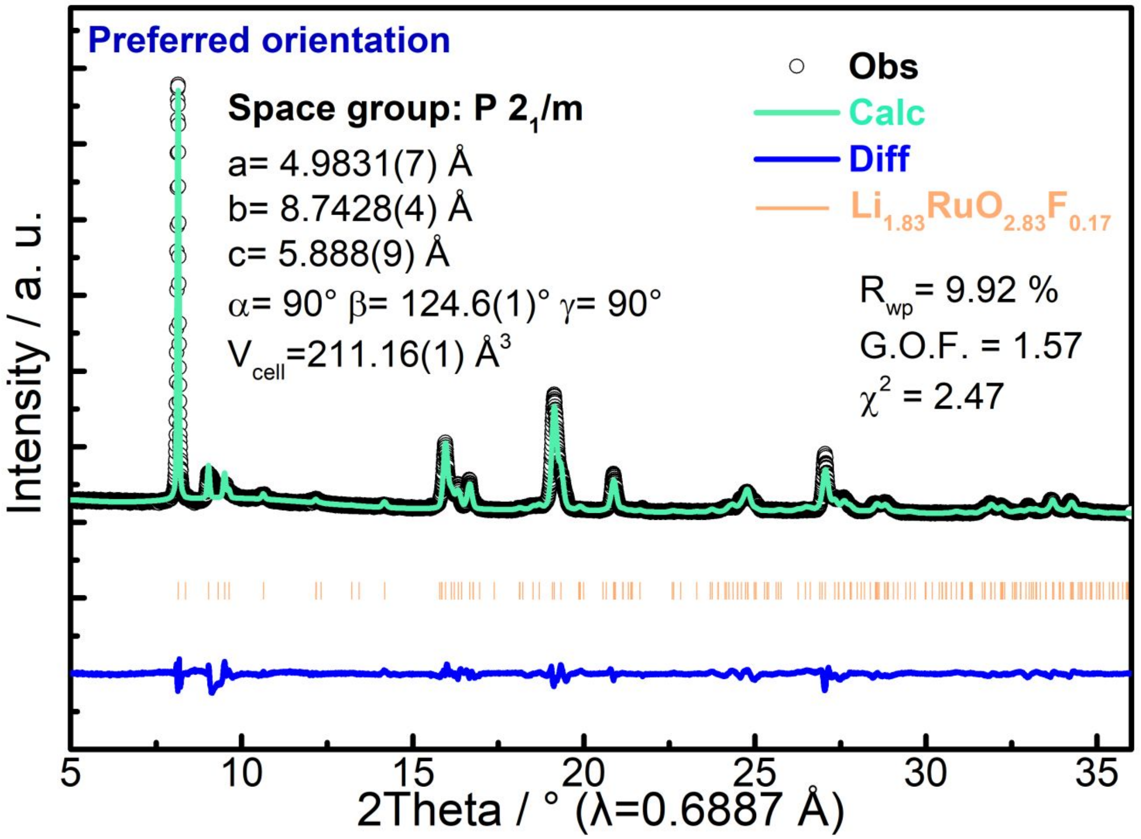

Figure S7. Rietveld refinement of $Li_{1.83}RuO_{2.83}F_{0.17}$ with synchrotron XRD patterns (λ=0.6887 Å) with preferred orientation considered.

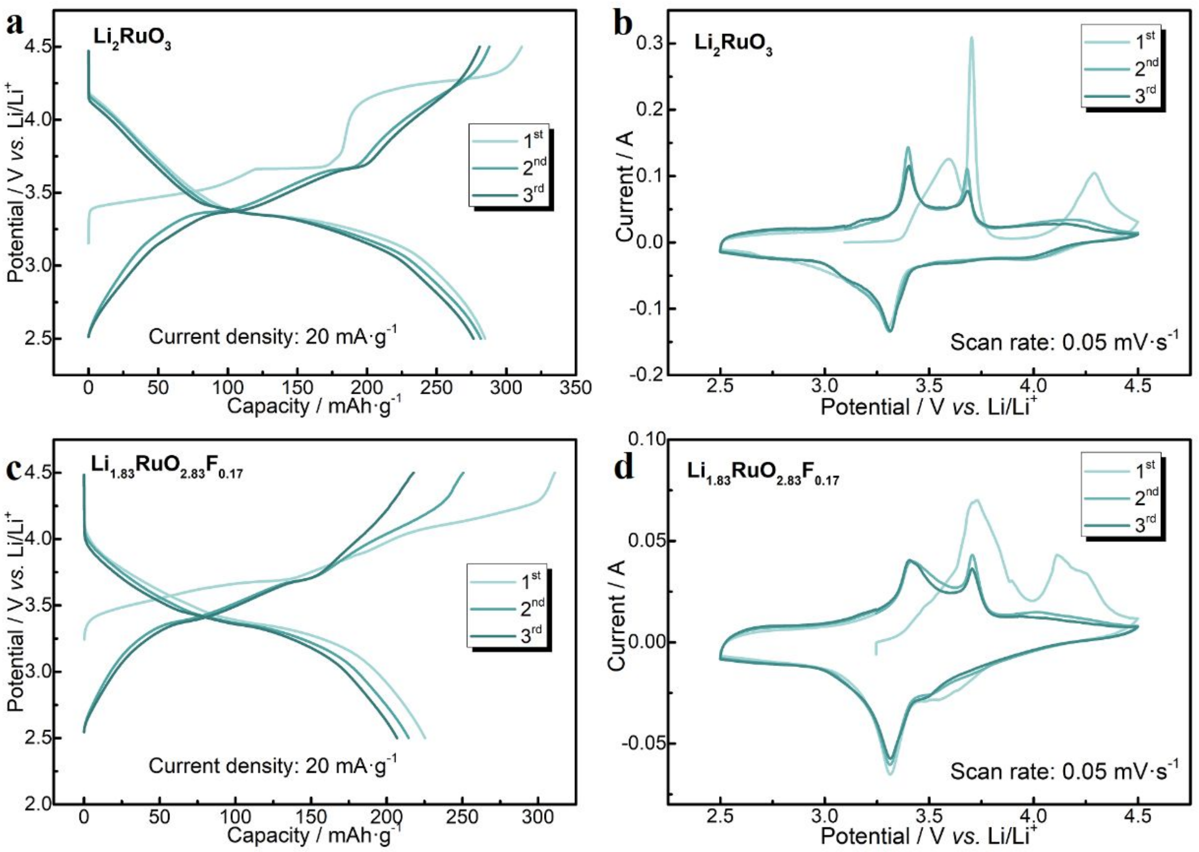

Figure S8. (a) The 1st-3th GCD and (b) CV curves of the Li_2RuO_3 electrode. (c) The 1st-3th GCD and (d) CV curves of the $Li_{1.83}RuO_{2.83}F_{0.17}$ electrode. The current density is 20 mA/g, the scan rate is 0.05 mV/s, and the potential window is 2.5-4.5 V vs. Li/Li^+.

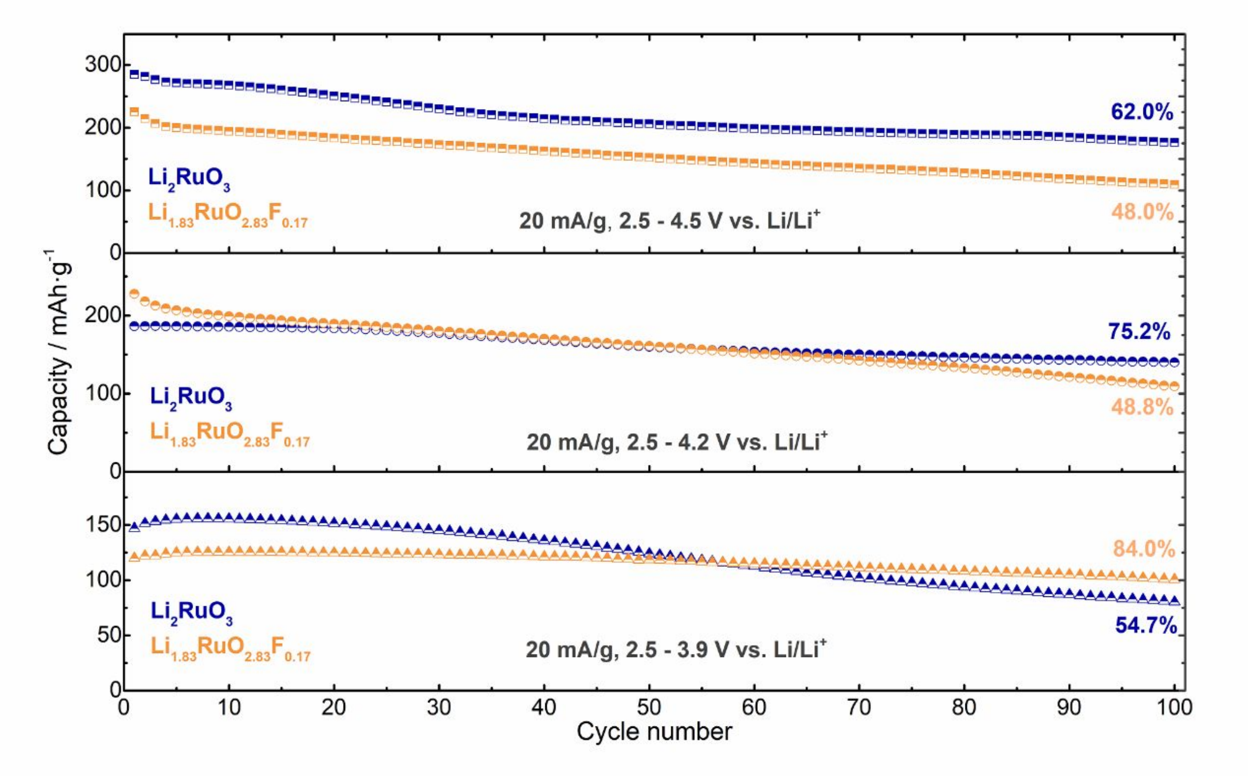

Figure S9. Cycling performances of Li_2RuO_3 and $Li_{1.83}RuO_{2.83}F_{0.17}$ electrodes at 20 mA/g within potential windows of 2.5-4.5 V, 2.5-4.2 V and 2.5-3.9 V vs. Li/Li^+ (from top to bottom).

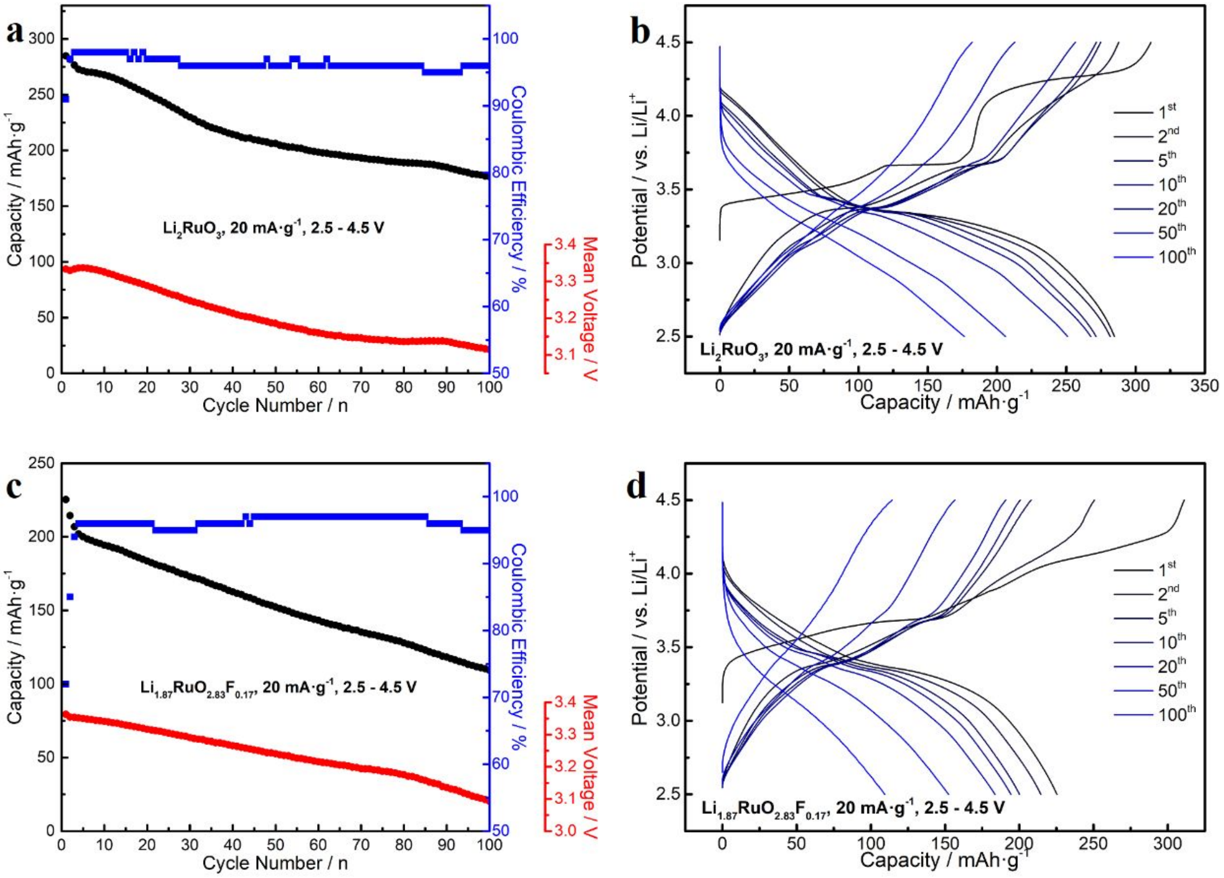

Figure S10. (a) Cycling performance of the Li_2RuO_3 electrode at 20 mA/g within the potential window of 2.5-4.5 V vs. Li/Li^+, and (b) corresponding GCD curves in different cycles. (c) Cycling performance of the $Li_{1.83}RuO_{2.83}F_{0.17}$ electrode at 20 mA/g within the potential window of 2.5-4.5 V vs. Li/Li^+, and (d) corresponding GCD curves in different cycles.

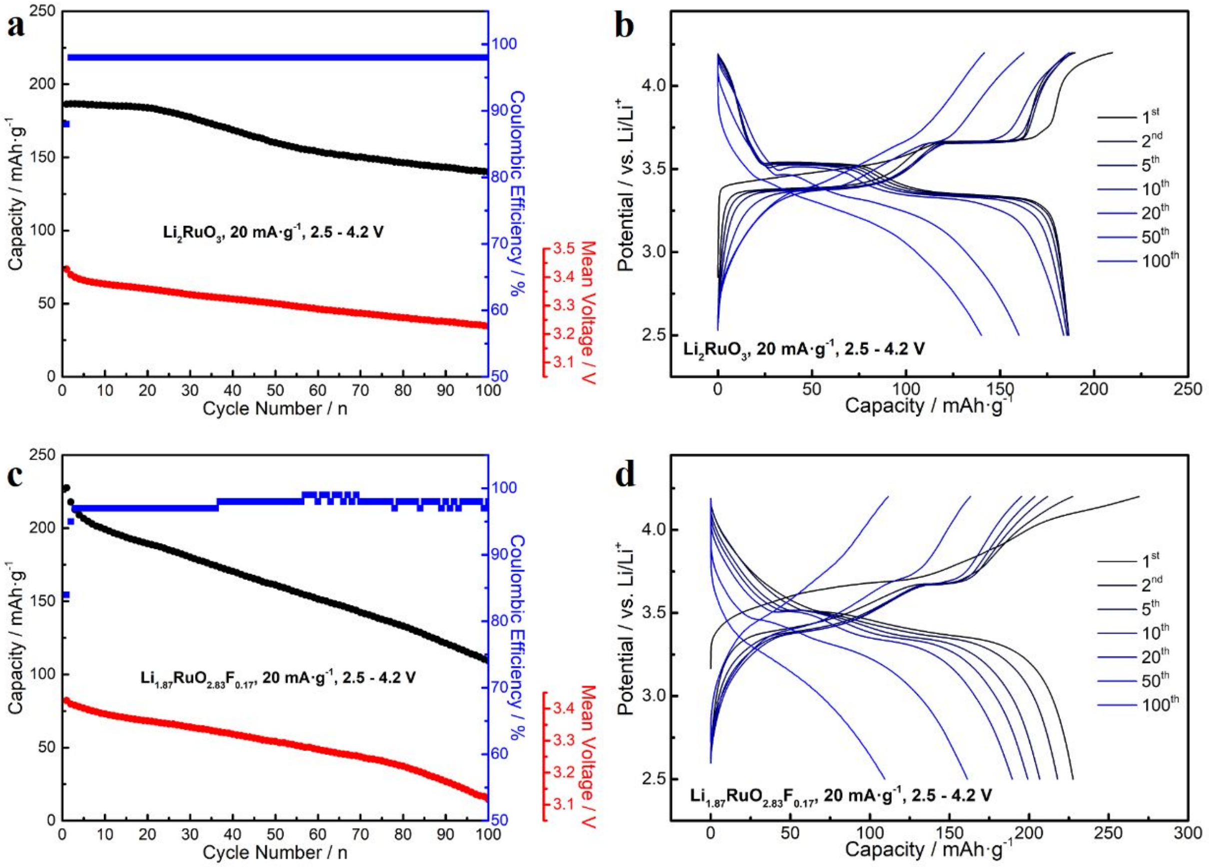

Figure S11. (a) Cycling performance of the Li_2RuO_3 electrode at 20 mA/g within the potential window of 2.5-4.2 V vs. Li/Li^+, and (b) corresponding GCD curves in different cycles. (c) Cycling performance of the $Li_{1.83}RuO_{2.83}F_{0.17}$ electrode at 20 mA/g within the potential window of 2.5-4.2 V vs. Li/Li^+, and (d) corresponding GCD curves in different cycles.

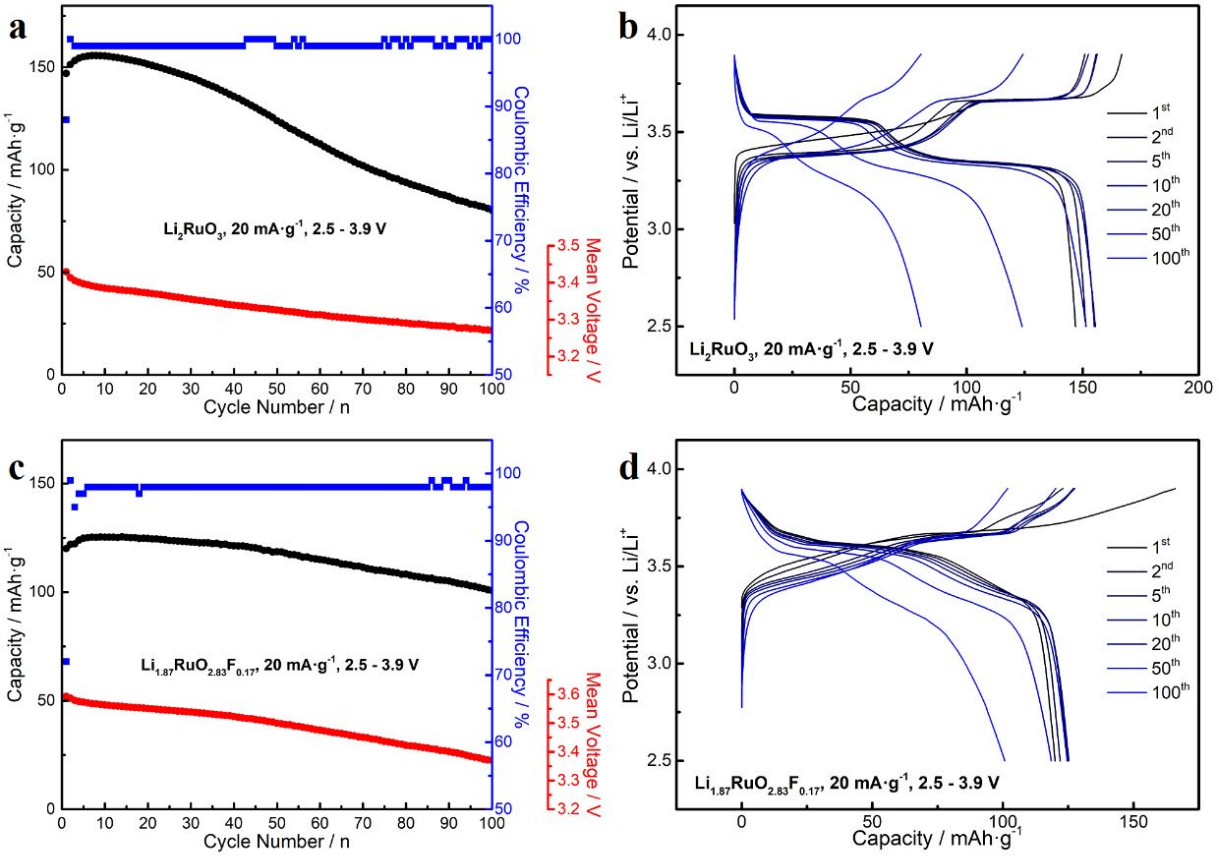

Figure S12. (a) Cycling performance of the Li_2RuO_3 electrode at 20 mA/g within the potential window of 2.5-3.9 V vs. Li/Li$^+$, and (b) corresponding GCD curves in different cycles. (c) Cycling performance of the $Li_{1.83}RuO_{2.83}F_{0.17}$ electrode at 20 mA/g within the potential window of 2.5-3.9 V vs. Li/Li$^+$, and (d) corresponding GCD curves in different cycles.

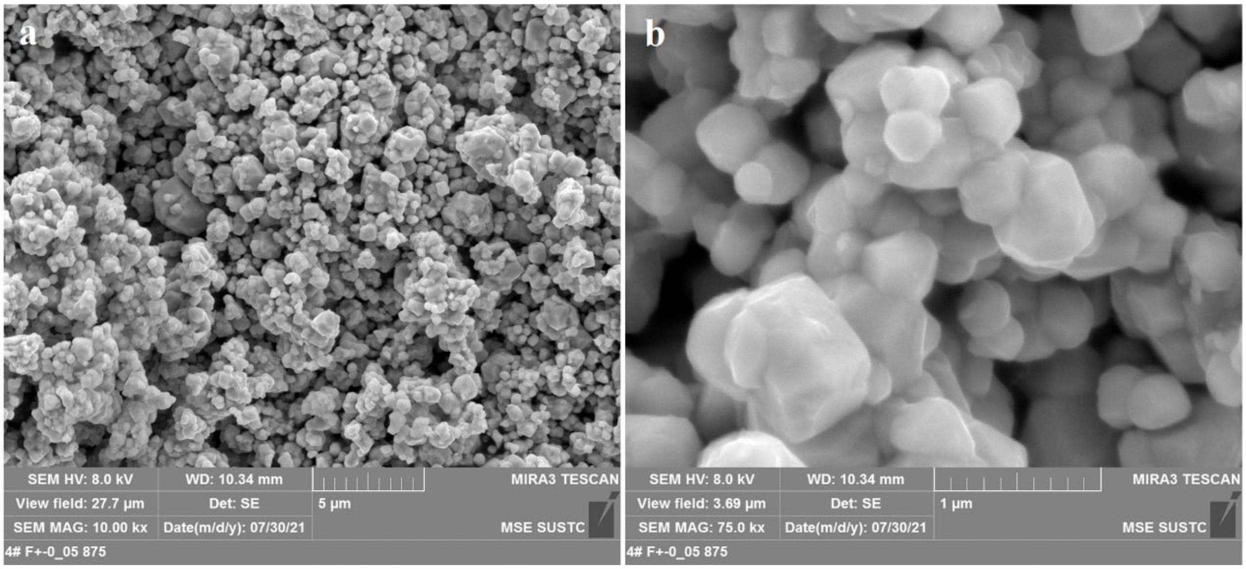

Figure S13. Scanning electron microscopy (SEM) images of the $Li_{1.95}RuO_{2.95}F_{0.05}$ prepared at 875 °C in air.

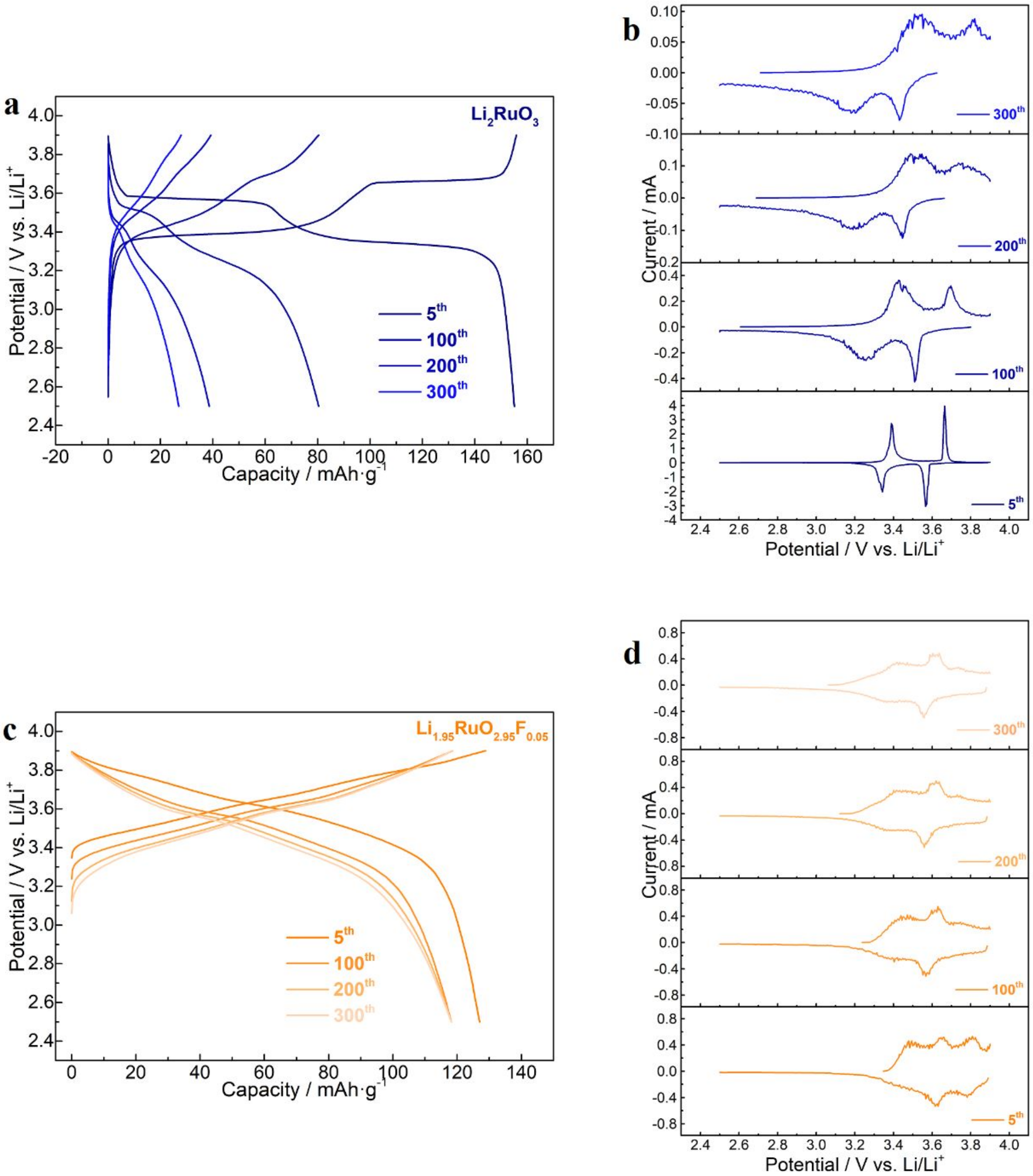

Figure S14. (a) GCD curves of the Li_2RuO_3 electrode within the potential window of 2.5-3.9 V vs. Li/Li^+ and (b) corresponding dQ/dV curves at the 5th, 100th, 200th, and 300th cycles. (c) GCD curves of the $Li_{1.95}RuO_{2.95}F_{0.05}$ electrode within the potential window of 2.5-3.9 V vs. Li/Li^+ and (d) corresponding dQ/dV curves at the 5th, 100th, 200th, and 300th cycles.

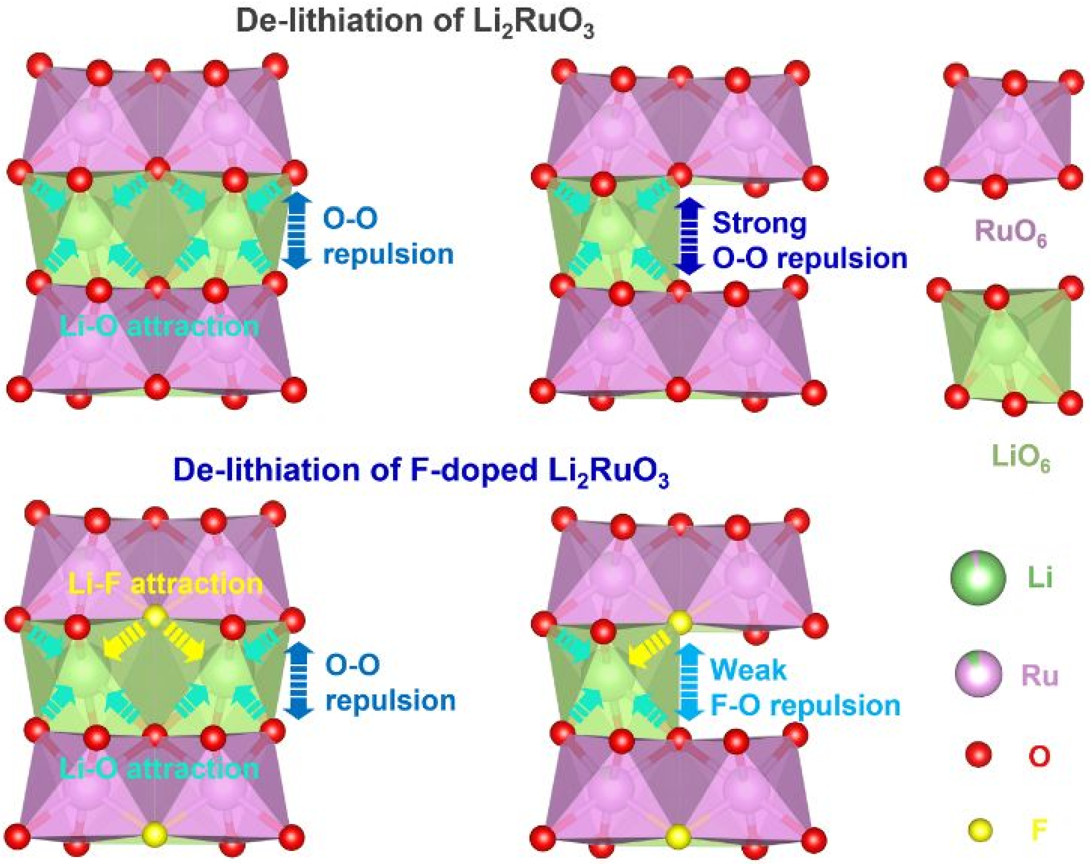

Figure S15. Schematic illustrations of interlayer interactions in pristine and de-lithiated Li_2RuO_3 (top) and F-doped Li_2RuO_3 (bottom). Views are from the [100] projection.

Table S1. Structural parameters of Li_2RuO_3 - no Li/Ru mixing and no preferred orientation

**Phase: Li_2RuO_3**		**Space group: $P\ 2_1/m$**			
Atom	Fractional coordinates			Multiplicity	Occupancy
	x	y	z		
Li1	0.7216(3)	0.25	0.9631(4)	2	1.00
Li2	0.2193(2)	0.0654(3)	0.4593(3)	4	1.00
Li3	0.7720(1)	0.25	0.5130(1)	2	1.00
Ru	0.2683(1)	0.0773(5)	0.9964(1)	4	1.00
O1	0.7408(5)	0.0654(3)	0.2519(8)	4	1.00
O2	0.7596(5)	0.1112(4)	0.8014(1)	4	1.00
O3	0.2815(5)	0.25	0.2347(5)	2	1.00
O4	0.3483(4)	0.25	0.8131(9)	2	1.00

Lattice parameters:

a=4.935(6) Å, b=8.7700(4) Å, c=5.8904(9) Å; α=90°, β=124.41(3)°, γ=90°;

$V_{unit\ cell}$=210.34(9) Å^3, R_{wp}=14.45 %, χ^2=2.46, G.O.F.=1.57

Table S2. Structural parameters of Li_2RuO_3 - only preferred orientation

**Phase: Li_2RuO_3**		**Space group: P 2_1/m**			
Atom	Fractional coordinates			Multiplicity	Occupancy
	x	y	z		
Li1	0.7216(3)	0.25	0.9631(4)	2	1.00
Li2	0.2193(2)	0.0654(3)	0.4593(3)	4	1.00
Li3	0.7720(1)	0.25	0.5130(1)	2	1.00
Ru	0.2654(4)	0.0777(2)	0.9925(3)	4	1.00
O1	0.7227(1)	0.0627(9)	0.2261(4)	4	1.00
O2	0.7467(3)	0.1021(4)	0.7785(7)	4	1.00
O3	0.2455(1)	0.25	0.2112(6)	2	1.00
O4	0.2879(9)	0.25	0.7585(6)	2	1.00
Lattice parameters:					
a=4.935(1) Å, b=8.7705(3) Å, c=5.8898(3) Å; α=90°, β=124.4(1)°, γ=90°;					
$V_{unit\ cell}$=210.32(8) Å^3, R_{wp}=11.02 %, χ^2=1.43, G.O.F.=1.20					

Table S3. Structural parameters of Li_2RuO_3 - both Li/Ru mixing and preferred orientation

**Phase: Li_2RuO_3**		**Space group: P 2_1/m**			
Atom	Fractional coordinates			Multiplicity	Occupancy
	x	y	z		
Li1	0.7102(4)	0.25	0.9656(8)	2	0.952(8)
Li2	0.2193(2)	0.0654(2)	0.4593(2)	4	1.00
Li3	0.7720(1)	0.25	0.5130(1)	2	1.00
Li4	0.2680(4)	0.0775(4)	0.9939(8)	4	0.023(6)
Ru1	0.7102(4)	0.25	0.9656(8)	2	0.047(2)
Ru2	0.2680(4)	0.0775(4)	0.9939(8)	4	0.976(4)
O1	0.7275(8)	0.0591(8)	0.2284(7)	4	1.00
O2	0.7552(1)	0.0984(6)	0.7730(1)	4	1.00
O3	0.2501(5)	0.25	0.2177(1)	2	1.00
O4	0.2766(4)	0.25	0.7535(7)	2	1.00
Lattice parameters:<br>a=4.935(1) Å, b=8.7707(1) Å, c=5.8898(6) Å; α=90°, β=124.40(6)°, γ=90°;<br>$V_{unit\ cell}$=210.33(3) Å^3, R_{wp}=10.70 %, χ^2=1.36, G.O.F.=1.16					

Table S4. Structural parameters of $Li_{1.83}RuO_{2.83}F_{0.17}$ - no Li/Ru mixing and no preferred orientation

**Phase: $Li_{1.83}RuO_{2.83}F_{0.17}$**		**Space group: P 2_1/m**			
Atom	Fractional coordinates			Multiplicity	Occupancy
	x	y	z		
Li1	0.7015(2)	0.25	0.9999(4)	2	0.915
Li2	0.2193(2)	0.0654(2)	0.4593(2)	4	0.915
Li3	0.7720(1)	0.25	0.5130(1)	2	0.915
Ru	0.2594(8)	0.0746(6)	0.9940(8)	2	1.00
O1	0.6822(7)	0.2562(2)	-0.0098(8)	4	0.943
O2	0.7575(5)	0.0792(2)	0.7547(5)	4	0.943
O3	0.2209(1)	0.25	0.2109(8)	2	0.943
O4	0.2744(5)	0.25	0.7518(9)	2	0.943
F1	0.6822(7)	0.2562(2)	-0.0098(8)	4	0.057
F2	0.7575(5)	0.0792(2)	0.7547(5)	4	0.057
F3	0.2209(1)	0.25	0.2109(8)	2	0.057
F4	0.2744(5)	0.25	0.7518(9)	2	0.057
Lattice parameters:					
a=4.9849(5) Å, b=8.7484(8) Å, c=5.886(9) Å; α=90°, β=124.57(5)°, γ=90°;					
$V_{unit\ cell}$=211.38(9) Å^3, R_{wp}=14.13 %, χ^2=5.01, G.O.F.=2.24					

Table S5. Structural parameters of $Li_{1.83}RuO_{2.83}F_{0.17}$ - only preferred orientation

**Phase: $Li_{1.83}RuO_{2.83}F_{0.17}$**		**Space group: $P\ 2_1/m$**			
Atom	Fractional coordinates x	y	z	Multiplicity	Occupancy
Li1	0.7015(2)	0.25	0.9999(4)	2	0.915
Li2	0.2193(2)	0.0654(2)	0.4593(2)	4	0.915
Li3	0.7720(1)	0.25	0.5130(1)	2	0.915
Ru	0.2572(5)	0.0798(1)	0.9886(5)	2	1.00
O1	0.6751(4)	0.0819(6)	0.0841(4)	4	0.943
O2	0.6961(8)	0.0842(1)	0.7605(5)	4	0.943
O3	0.2306(7)	0.25	0.2171(1)	2	0.943
O4	0.2701(1)	0.25	0.7291(1)	2	0.943
F1	0.6751(4)	0.0819(6)	0.0841(4)	4	0.057
F2	0.6961(8)	0.0842(1)	0.7605(5)	4	0.057
F3	0.2306(7)	0.25	0.2171(1)	2	0.057
F4	0.2701(1)	0.25	0.7291(1)	2	0.057

Lattice parameters:

a=4.9831(7) Å, b=8.7428(4) Å, c=5.888(9) Å; α=90°, β=124.6(1)°, γ=90°;

$V_{unit\ cell}$=211.16 (1) Å^3, R_{wp}=9.92 %, χ^2=2.47, G.O.F.=1.57

Table S6. Structural parameters of $Li_{1.83}RuO_{2.83}F_{0.17}$ - both Li/Ru mixing and preferred orientation

**Phase: $Li_{1.83}RuO_{2.83}F_{0.17}$**	**Space group: P 2_1/m**				
Atom	Fractional coordinates			Multiplicity	Occupancy
	x	y	z		
Li1	0.7015(2)	0.25	0.9999(4)	2	0.649(6)
Li2	0.2193(2)	0.0654(2)	0.4593(2)	4	0.915
Li3	0.7720(1)	0.25	0.5130(1)	2	0.915
Li4	0.2581(3)	0.0789(8)	0.9891(1)	4	0.132(7)
Ru1	0.7015(2)	0.25	0.9999(4)	2	0.265(4)
Ru2	0.2581(3)	0.0789(8)	0.9891(1)	4	0.867(3)
O1	0.6722(2)	0.0660(1)	0.0721(3)	4	0.943
O2	0.7317(1)	0.1005(1)	0.7583(7)	4	0.943
O3	0.2586(6)	0.25	0.2256(2)	2	0.943
O4	0.2540(1)	0.25	0.7228(6)	2	0.943
F1	0.6722(2)	0.0660(1)	0.0721(3)	4	0.057
F2	0.7317(1)	0.1005(1)	0.7583(7)	4	0.057
F3	0.2586(6)	0.25	0.2256(2)	2	0.057
F4	0.2540(1)	0.25	0.7228(6)	2	0.057

Lattice parameters:

a=4.9811(5) Å, b=8.7439(4) Å, c=5.8873(9) Å; α=90°, β=124.58(6)°, γ=90°;

$V_{unit\ cell}$=211.10(8) Å^3, R_{wp}=8.78 %, χ^2=1.94, G.O.F.=1.39

Table S7. Structural parameters of $Li_{1.95}RuO_{2.95}F_{0.05}$ - both Li/Ru mixing and preferred orientation

**Phase: $Li_{1.95}RuO_{2.95}F_{0.05}$**		**Space group: P 2_1/m**			
Atom	Fractional coordinates x	y	z	Multiplicity	Occupancy
Li1	0.7194(8)	0.25	1.0102(6)	2	0.518(1)
Li2	0.2388(3)	0.0701(4)	0.4689(5)	4	0.975
Li3	0.7940(3)	0.25	0.5330(1)	2	0.975
Li4	0.2729(5)	0.0855(4)	1.0205(4)	4	0.228(5)
Ru1	0.7194(8)	0.25	1.0102(6)	2	0.457(1)
Ru2	0.2729(5)	0.0855(4)	1.0205(4)	4	0.771(5)
O1	0.6857(1)	0.0610(1)	0.2565(9)	4	0.983
O2	0.7867(1)	0.0269(5)	0.7806(1)	4	0.983
O3	0.2329(7)	0.25	0.2915(2)	2	0.983
O4	0.2670(1)	0.25	0.7795(9)	2	0.983
F1	0.6857(1)	0.0610(1)	0.2565(9)	4	0.017
F2	0.7867(1)	0.0269(5)	0.7806(1)	4	0.017
F3	0.2329(7)	0.25	0.2915(2)	2	0.017
F4	0.2670(1)	0.25	0.7795(9)	2	0.017

Lattice parameters:

a=5.0440(2) Å, b=8.7245(4) Å, c=5.8961(1) Å; α=90°, β=124.88(8)°, γ=90°;

$V_{unit\ cell}$=212.83(4) Å^3, R_{wp}=4.93 %, χ^2=1.32, G.O.F.=1.15

Supplementary References

(1) Toby, B. H.; Von Dreele, R. B. GSAS-II: the genesis of a modern open-source all purpose crystallography software package. *J. Appl. Cryst.* **2013**, *46*, 544-549.
